# Response to Damianos—Anthropocene angst: Authentic geology and stratigraphic sincerity

**DOI:** 10.1177/03063127251343046

**Published:** 2025-06-08

**Authors:** Colin N Waters, Jan Zalasiewicz, Martin J Head, Georg N Schäfer, Francine MG McCarthy, Simon D Turner

**Affiliations:** 1 School of Geography, Geology and the Environment, University of Leicester, Leicester, UK; 2 Department of Earth Sciences, Brock University, St. Catharines, ON, Canada; 3 Max Planck Institute of Geoanthropology, Jena, Germany; 4 University College London, London, UK

**Keywords:** Anthropocene, formalization, media, politics

## Abstract

Damianos provides his views on the significance of the March 2024 decision by the International Commission on Stratigraphy (ICS) to reject the proposal of the Anthropocene Working Group (AWG), the body we represent, to formalize the Anthropocene as a series/epoch of the Geological Time Scale. He draws upon ‘four years of ethnographic observation’ of the AWG, over which time this body provided him with access to its meetings and discussions. Given this access, the numerous misrepresentations within his article warrant redress. Ultimately, his conclusions mimic claims of influential figures within the governing bodies of the stratigraphic process: that the AWG were attempting to formalize the Anthropocene for political reasons and subvert the process through use of the media, and that the proposed definition was based upon claims about the future and not the past geological record. We refute those accusations, and emphasize that the proposed Anthropocene epoch, based on scrupulous and detailed analysis of the stratigraphic record, demonstrates striking and transformative Earth System change driven by the mid-20th century ‘Great Acceleration’ of human activities.

The Anthropocene Working Group (AWG) of the Subcommission on Quaternary Stratigraphy (SQS), a constituent body of the International Commission on Stratigraphy (ICS), was established in 2009 to determine if there was merit in formalizing the Anthropocene as a unit of the geological time scale. If so, the AWG were tasked with submitting a proposal to SQS: (a) explaining why the Anthropocene should be at series/epoch rank, hence terminating the Holocene that accounts for the past 11,700 years, (b) determining when the Anthropocene should broadly commence and (c) then providing a precise definition through recognition of a specific Global boundary Stratotype Section and Point (GSSP or ‘golden-spike’) in a section that exemplifies the key stratigraphic markers for long-distance correlation.

Funding for the analytical phase of the AWG’s research was largely provided by the Haus der Kulturen der Welt (HKW) in Berlin as part of a transdisciplinary, scientific, and cultural exploration of the Anthropocene. The ~850,000 euros (not 1 million euros stated by Damianos, 2025, p. 18), available in 2020–2023, also covered organization of meetings to contribute to discourse programmes, publication projects and exhibitions at HKW (Rosol et al., 2023; Scherer, 2022).

The data-rich 191-page submission to SQS on 31st October 2023 comprised three parts (subsequently made available openly): an Executive Summary (Waters et al., 2024a), the stratigraphic context and justification for rank (Waters et al., 2024b), and the description of the proposed GSSP and supporting sections which would, if accepted, have provided the formal definition for the base of the Anthropocene and hence constrained the age of its onset (Waters et al., 2024c). The SQS was tasked with discussing the proposal in detail and voting on it, but neither was concluded within the statutory rules to which SQS was bound. Instead, the ICS hurriedly and without input from the SQS chair rejected the AWG proposal and this decision was upheld with similar haste by the Executive Committee of its parent body, the International Union of Geological Sciences (IUGS, 2024).

In Damianos’s assessment of the process that ultimately led to the rejection of the proposal, important errors and biased interpretations may be summarized as follows:

(1) Suggesting that the AWG followed a different process to analyse the Anthropocene than is typical of other chronostratigraphic units;
(2) Promoting arguments raised by those opposed to the work of the AWG without critical appraisal;
(3) Claiming that the AWG was driven by political motives rather than objectivity and scientific evidence;
(4) Claiming that AWG used the media to attempt to subvert the rules of the governing stratigraphic body and to gain popular support;
(5) Incorrect statements on the nature of the SQS vote that follow unquestioning acceptance of the ICS/IUGS narrative;
(6) Pejorative comments on the motives of the AWG Chair based upon a media interview that is selectively quoted.

## Perceived erroneous methodologies by AWG

To underpin one part of his argument, Damianos (2025, p. 4) quotes Pottage (2019, p. 154): ‘[W]hat makes the Anthropocene unusual is that ‘instead of beginning with the fossil and eliciting context from it, one begins with context and finds the Leitfossil for that context’.’ The cited passage misunderstands how the process works. The decision to define a chronostratigraphic unit never starts by deciding upon a particular marker for its base. In most cases, it is recognition of a context of change to the geology of the planet that indicates the necessity for a boundary, and once that necessity is agreed, it is the responsibility of the relevant formal stratigraphic subcommission, typically through establishing a specific task group, to then look for appropriate evidence in the strata that might provide an effective boundary marker. Increasingly, that proxy evidence for younger parts of the geological time scale is through geochemical signals as these can constitute clearer and more isochronous signatures than fossils (Waters et al., 2018). Damianos oversimplifies in stating that there is reliance on ‘established techniques of (primarily) palaeontological observation’. In fact, this is not the case for the Quaternary Period, nor any of its constituent epochs and ages, which are defined by GSSPs placed at lithological, geochemical, or palaeomagnetic markers. The AWG assembled a range of lithostratigraphic, biostratigraphic and chemostratigraphic evidence in essentially classical format, employing ~100 stratigraphic markers (Kuwae et al., 2024; Waters et al., 2023), significantly more than the six markers associated with the Holocene Epoch GSSP in the NGRIP2 core from the Greenland Ice Sheet (M. Walker et al., 2008).

Damianos (2025, p. 9) states: ‘The contested nature of the Anthropocene formalization effort is an effect of the Anthropocene Working Group’s *appropriating* interpretive techniques, such as palaeontology and the fossil, toward a *speculative* account of life on Earth.’ This insinuates that the Anthropocene is not underpinned, or indeed predicated, by legitimate geology and that AWG are *appropriating* methods used by teams studying other parts of the geological column, pretending that they apply to deposits of the recent past. It is important to realize that standard protocols for defining any GSSP have been outlined by Remane et al. (1996) and they apply to deposits of any age. The AWG followed these guidelines when selecting a suitable GSSP (see Waters et al., 2023) specifically because the methodologies used in deep-time stratigraphic analysis were seen as equally applicable to recently deposited strata, and with the view that the Anthropocene should not be treated as an exception. By repeating the term as follows ‘The AWG was authentic because it *appropriated* geology to moral and normative claims’ (Damianos, 2025, p. 11) he conveys a message that we are only using geology as part of some perceived moral campaign, and belittles the research carried out by AWG to study the geological record of the Anthropocene. This contention is repeated as ‘Yet the AWG was arguably less concerned with a designation of the past than with a warning about the future’, which misrepresents the AWG’s actions and intentions.

Furthermore, in describing the AWG’s approach as *speculative*, Damianos might be taken as suggesting that the work of the AWG is based on conjecture rather than objective evidence. This statement denigrates the 14 years of extensive data collection that involved many scientists, often underpinned by research pre-dating the AWG investigations, to reach our conclusion. We note that unlike other parts of the geological time scale, the Anthropocene as characterized by the AWG has not only an extensive and detailed geological record, but one that is correlated with and supported by quantitative measurements and human observation. The Anthropocene provides a less speculative account of life and process on Earth than any interpretation of deep-time planetary history!

The term ‘appropriation’ is repeated again on page 10: ‘In appropriating palaeontological techniques to advance a forward-looking account of Earth, the AWG shifted its gaze from 4.5 billion years of deep time planetary history to oracular prediction’, once more misrepresenting the work of the AWG. The proposal submitted for formalization of the Anthropocene is based on geological records that have already accumulated. They are not future predictions of what strata will be deposited: they already exist! Where the AWG considered it necessary to discuss future scenarios was to counter the common accusation that the Anthropocene is too short and of too little consequence to be a geological time unit, for example from Stanley Finney, the then Secretary General of IUGS in the *New York Times*, 17 Dec. 2022 that ‘the Anthropocene is “a blip of a blip of a blip”’ (Zhong, 2022). This ignores the AWG’s demonstration that the Anthropocene, while brief to date, has *already* irreversibly changed the Earth from its Holocene state. Assessment of climate modelling (see Summerhayes et al., 2024) and changes to biodiversity (see Williams et al., 2024) show that the Anthropocene will have geologically long-term consequences; it is no blip. But its stratigraphic record to date is already extensive, distinctive, documented with unparalleled precision, and highly amenable to formal definition.

Damianos (2025, p. 11) states that SQS ultimately rejected the AWG proposal ‘on the basis of preserving the sincerity of geological methodology’. This is simply wrong. The methodology pursued by the AWG and the resulting extensive database formed no part of the reasons given for rejecting the Anthropocene. The rejection (by ICS, not SQS) occurred *despite* the proposal precisely following standard stratigraphic practice and methodologies. Given that the voting occurred after minimal discussion of the scientific evidence, it can only be inferred that the rejection was based upon ideology—a preconceived notion of the minimum duration for an epoch and not from the geological evidence in the proposal. The question of sincerity here is turned on its head.

### Unquestioning promotion of concepts contrary to those forwarded by AWG

In the context of the AWG providing a definition for a specific point for the base of the Anthropocene, Damianos (2025, p. 8) states ‘that any rock below that layer would not be “of the Anthropocene”. Archaeologists, anthropologists, and anyone with a passing interest in anthropogenic planetary interventions prior to the mid-20th century were *justifiably* stunned by the *ignorance* this gesture entailed.’ Damianos here selectively accepts opinions critical of the AWG proposal, describing the approach as ignorant in lacking consideration of human impacts prior to 1952. We emphasize that geological deposits associated with human impacts prior to the Anthropocene are not ignored: They are part of the Holocene (and indeed extend yet further back into the Pleistocene). The AWG has repeatedly asserted—as Damianos must be aware—that the proposed Anthropocene epoch does *not* equate with ‘all detectable anthropogenic impacts’ which do range back many millennia and have been described at considerable length in numerous AWG publications. Indeed, part of the characterization of the Holocene, distinguishing it from earlier interglacials, is that it has an abundant archaeological record (M. Walker et al., 2008). Damianos’s assertion suggests that assigning something to the Holocene gives it diminished status. *All* adjacent geological time units are separated by a specific isochronous boundary, and strata are assigned to one side of the boundary or the other (Head et al., 2023, p. 233). This provides specific labels that geologists find useful, to allow packages of strata to be assigned to a particular geological time interval or another. There has never—until now—been any implication that one geological time unit was somehow better or more desirable than another.

Damianos then goes on to support the case for an alternative usage of the term, the ‘Anthropocene event’ that has been recently promulgated principally by Gibbard and colleagues (e.g. Gibbard et al., 2022) as a counter to the AWG work. He states ‘a geological *event*, by comparison, acknowledges the diachronous and multiple, simultaneous, regional *episodes* that collectively point to an occurrence that cannot be reduced to a singularity. The growing intensity of anthropogenic influence on planetary dynamics is acknowledged without anchoring it to a particular historical moment, without situating the Anthropocene within a single geo-anthropological narrative.’ In fact the ‘Anthropocene event’ is neither geological in the strict sense nor an ‘event’, at least in normal Quaternary usage. The AWG has published detailed responses to this ‘event’ (e.g. Head, Steffen, et al., 2022; Head et al., 2023; Waters et al., 2022), but Damianos neglects to mention these or the arguments they advance, opting to take the ‘event’ approach at face value and to cite the papers that support it. The central issue is that a concept, of course, might be developed to encompass all human impacts through all time—but the ‘event’ formulation that does this is very different from the Anthropocene epoch concept as postulated by Paul Crutzen and developed by the AWG that the Anthropocene terminates the Holocene. There is no reason to give it the same name, ‘Anthropocene’—except as a means to undermine formalization.

Damianos’s comment shows, too, a lack of understanding of how an ‘event’ is usually used in stratigraphy, and especially for the Quaternary—not as a vague diachronous phenomenon, but as an abrupt happening that can be used as a near-instantaneous time marker to correlate strata on varying geographical scales. An event is not the same as an episode in this technical sense, as confused by Damianos in the above quote. An episode is a prolonged and potentially diachronous phenomenon, and is in fact the term that more correctly describes the Gibbard et al. concept. The ‘event’ proposal deliberately ignores the overwhelming scientific evidence of the Great Acceleration (e.g. Head, Zalasiewicz, et al., 2022; Steffen et al., 2007, 2015) which underpins the AWG selection of a mid-20th century start for the Anthropocene and which geologically is strikingly tightly defined, so that an individual year, 1952, can be identified on a global scale (Kuwae et al., 2024; McCarthy et al., 2025). Damianos, extraordinarily, fails to mention the Great Acceleration in his article, by this omission supporting the most spurious argument used against the AWG proposal. He also fails to appreciate the contradiction that comes from a later quote from Edwards et al., 2017): ‘[P]recise boundaries are the basis for defining geologic time, a prerequisite for the correlation of abiotic and biotic *events* and the understanding of the rates and timing of biological and geological processes on our planet.’ These authors, who elsewhere advocate using ‘events’ to describe vague and diachronous phenomena are in this instance actually using the term appropriately, and as applied by the AWG. Geologically abrupt events are in practice commonly used to guide the position of GSSPs for chronostratigraphic units, and Waters et al. (2022; see also Kuwae et al., 2024) noted the numerous distinct events that cluster around the mid-20th century where they provide ample means of and justification for identifying a GSSP for the Anthropocene.

## Perceived political motives in the work of the AWG

The assertion that ‘The Anthropocene formalization effort was the result of attempts to articulate political and normative assertions on the register of geoscientific fact …’ (Damianos, 2025, p. 2) misrepresents the work of the AWG in a fashion comparable to the persistent charges that the AWG analysis of the Anthropocene is politically, not scientifically, motivated, including by the then IUGS Secretary General (Finney & Edwards, 2016), repeated by Finney in the *New York Times*, December 17 2022 that ‘Stanley C. Finney … fears the Anthropocene has become a way for geologists to make a ‘political statement’’ (Zhong, 2022).

The goal of the AWG has always been to assess the scientific evidence and determine whether it warrants establishment of a new geological epoch. It is true that the implications of these data include recognition of significant deterioration in the state of our planet, and individual AWG members have been invited to discuss future societal concerns at conferences and in the media. However, this is not the driving force of the AWG, but is simply one consequence of growing awareness of planetary repercussions as revealed by the scientific analysis (e.g. Klingan et al., 2025, p. 5). An accusation that the AWG is politically motivated is akin to claiming that the vast number of climate scientists that have reached a consensus on anthropogenic climate change have done so for political reasons and not to report scientific evidence. Damianos makes no attempt in his article to consider whether those involved in arguing publicly against the AWG proposal were doing so for political reasons, or to explore why they persistently refused to engage with the data. Further, he states ‘the term was first announced as an explicitly political statement’ (Damianos, 2024, p. 2)—and we assume that this relates to the description of the term by Crutzen and Stoermer (2000) and Crutzen (2002). These publications first drew the world’s attention to a term coined separately by these two authors that summarized a range of Earth System changes that were already then leading to the conclusion that the planet had left the envelope of variability characteristic of the Holocene. This was a synthesis of scientific data, rather than a call to arms to save the planet.

## Perceived use of media by AWG to subvert due process

The article reports the ‘accusation that the AWG was primarily concerned not with “science”, but with performing what one interlocutor described as “big announcements to the press”’ (Damianos, 2025, pp. 2–3). Damianos did not name the source of this defamatory comment, nor made any attempt to disagree with it. We recognize this as an accusation of the kind often made by those wishing to see the proposal fail, such as by the then IUGS Secretary General who has stated ‘the leaders of the working group are having too much fun publishing and getting publicity’ (Carey, 2016, p. 3909) and more recently ‘The Anthropocene epoch was pushed through the media from the beginning—a publicity drive,’ (Voosen, 2024). This accusation is countered by the reality that the AWG has simply been reactive to the many requests for comment by journalists, such as Voosen. In effect the AWG is being criticized for the public interest in its work. The interest thus stimulated in the previously publicly obscure process of stratigraphic labelling has enabled this area of science to be discussed more openly. Such public outreach is now regarded as among the goals of any scientist, not least by universities and funding bodies. AWG members have always been generous with their time when receiving such approaches from the media, as they were too in discussing their work with Damianos. It is surely laudable to break from the old attitude that scientists should work in their ‘ivory towers’ heedless of any need for public accountability. Not only do the ICS statutes (International Commission on Stratigraphy [ICS], 2017) not prohibit or advise against discussion of stratigraphic science with the media, they avow as a principal objective ‘the promotion of education in stratigraphic methods, and the dissemination of stratigraphic knowledge’. Nevertheless, the ICS and IUGS executives criticized open discussion of the Anthropocene topic by the AWG—while simultaneously providing repeated denigratory statements about the AWG to the media. Damianos fails to find this contradictory.

Damianos provides a lengthy narrative on the events of the STRATI 2023 congress in Lille, France, convened by Thomas Servais. At that meeting, there was considerable media interest to hear the AWG announcement of which candidate site had been chosen—though the statement by Damianos on page 14 that ‘there were almost as many journalists present as there were stratigraphers and geologists’ is hyperbole. The few media people who applied to attend personally were admitted to the meeting under instruction from STRATI organizers not to speak with the AWG, the only group singled out for this ban. To speak with AWG members, interviews had to be conducted outside of the building. Contrary to Damianos’s statement that ‘according to the organizers, they had not obtained the appropriate license to film conference proceedings’, the film crew had requested a licence in advance of the conference and were happy to meet any imposed conditions, but the organizers decided not to issue the licence. Furthermore, the ICS Executive had requested that a press conference scheduled for April 2023 following the conclusion of AWG voting be postponed so that it could be given at STRATI 2023, following announcement to conference attendees. STRATI 2023 subsequently rescinded the agreement and banned AWG from holding a press conference at the conference venue: hence, the need to rearrange the meeting to the conference room of a nearby hotel. The announcement of the AWG’s proposed candidate site was made at a joint press conference with the Max Planck Society to complete a contractual obligation with the body that funded the analysis of the candidate sites: This marked the conclusion of the 10-year Anthropocene project of HKW and the Max Planck Institute for the History of Science (Rosol et al., 2023; Schäfer, 2023).^1^

There is further bias in the statement ‘The cart came before the horse: An announcement had been made as if the GSSP had been decided, when it had yet to be formally approved by the decision-making committee of the SQS and ICS.’ Here, Damianos, who was present at the press conference, ignores the clarification provided there as to why the meeting was being held and how it represented simply another stage in the process and not a formal approval. It is normal for a candidate GSSP to be made known prior to the beginning of formal discussion and voting, for this allows the widest feedback on the proposal and the data. For instance, GSSPs proposed for the subdivision of the Holocene were published explicitly in a discussion paper (M. J. C. Walker et al., 2012), with the subsequent vote and ratification only being finalized several years later, in 2018 (M. Walker et al., 2018).

Damianos states that in their keenness for publicity, the AWG were responsible for ‘side-stepping the formal procedures established to do so’ (p. 16), without explaining what formal procedures were broken. Rather than querying what ICS statutes were violated by AWG (none were), he preferred to follow the ICS narrative unquestioningly, even failing to mention that ICS was documented to have broken its own statutes in the eagerness to reject the proposal (Turner et al., 2024; Zhong, 2024), which is arguably a more newsworthy issue and certainly one that would have been noted in any impartial account. He concluded that ‘the AWG sought to refute the priority of the decision-making procedures those bodies have established, by developing close ties with journalists and mass-media outlets, in an attempt to pre-determine the outcome of the Anthropocene vote “in the court of public opinion”’ Damianos (2025, p. 17). This suggests that the AWG purposely courted supposed media contacts to subvert the formal process: an allegation that one can only describe as scurrilous.

## A biased assessment of the SQS vote

Damianos (2025, p. 5) is incorrect to state that SQS Executive members Jan Zalasiewicz and Martin Head, who were also members of the AWG, ‘were forbidden from voting on the final proposal, due to a perceived conflict of interest’. In fact, both Zalasiewicz and Head refused to submit a vote, given that the voting process had not been concluded in accordance with official ICS statutes (ICS, 2017), and hence asserted that the vote was not legitimate. P.L. Gibbard, as then Secretary General of ICS, was equally in conflict of interest given his prolonged and public campaign against the AWG proposal while dictating the terms under which it could be discussed within SQS, but nevertheless still voted (against the proposal). Indeed there is no conflict of interest provision within the ICS statutes.

### Personal attack on the motives of the AWG Chair

Damianos reserves specific criticism for the AWG Chair in asserting that he was less concerned with rigorous scientific analysis and more with trying to establish AWG’s position on the high moral ground. ‘To reject a formal Anthropocene Series/Epoch, argued AWG Chair Colin Waters, would be “almost akin to someone saying climate change doesn’t exist” (Ley [sic], 2023).’ Significantly, he omits the previous sentence which provides context: ‘If the proposal is rejected, however, it could appear as if we are overlooking the outsized influence of humans over the past few decades, says Waters’ (Ly, 2023). The omitted sentence offers a view by Waters that there could be concern as to how this decision might be perceived. Damianos has taken the quote out of context apparently for dramatic effect, but one has to ask how he can so confidently conclude ‘Clearly that is not true’. As a result, he asserts that Waters felt the proposal ‘should be accepted, if not on the basis of scientific rigour or agreement with previous decisions concerning amendments to the Geologic Time Scale, then at the very least out of a moral obligation to acknowledge anthropogenic climate change’ (Damianos, 2025, p. 13). To state that a scientist, whose reputation is based upon the quality of research he is involved in, cares less about scientific rigour than some perceived goal, is defamatory.

In the context raised, the AWG has presented an extensive body of scientific data that evidences an abrupt global transition of the Earth System, including the climate system, in the mid-20th century. This may be contrasted with ignoring the evidence of the Great Acceleration and its consequences, and replacing it by a model of apparently slow gradual change to a planet from human impacts ranging back for tens of millennia (as in Gibbard et al., 2022; see Turner et al., 2024 for a critique of its scientific basis). This indeed seems comparable to ignoring the scientific evidence of anthropogenic climate change, and stating that this doesn’t exist because the climate has always changed. We note that one of the SQS voting members, who voted against the AWG proposal, refuses to accept the role of humans in driving contemporary climate change (Marks, 2025).

## Conclusion

Damianos’s conclusion that ‘Members of the AWG are therefore self-assertively virtuous, at least as their own self-presentation is concerned, even if the claim they were asserting has been rendered, through popular vote, scientifically false’ wrongly claims the decision of a body of just 22 scientists as being some form of popular vote, and that normative science can be reduced to a ‘true’ or ‘false’ outcome. Some members of SQS had expressed open hostility to a formalized Anthropocene from the outset, whilst others with no specific interest in the subject (or uncomfortable with the fraught situation that developed) may have taken their lead from the long-standing hostility to the AWG expressed by the authorities running their parent bodies, the ICS and IUGS. The result of the vote does not render the AWG submission any more false than the longstanding early rejection of plate tectonics—while the voting process itself has been openly questioned. The author makes no attempt to question the legitimacy of that the SQS vote, despite widely available reference to irregularities, again reflecting bias throughout his narrative. Damianos has taken every opportunity to criticize the methodologies and motives of the AWG, without equal questioning of the operations of the organisations overseeing the process. We are not arguing that the work of the AWG is above criticism, but that its evaluation should be fair and balanced.

## Notes

1. Damianos (2024, p.16) claims there had been a livestream to HKW during the joint press event. The public online event was broadcast both from Lille and the Max Planck Society’s Harnack Haus in Berlin. While the first part of the event largely consisted of MPI and former HKW members reflecting on the 10-year collaboration with the AWG in Berlin, the second part of the event concluded with the AWG’s announcement of the proposed candidate site. At both broadcasting locations, the public was invited.
